# Structural Properties of Cruciferin and Napin of *Brassica napus* (Canola) Show Distinct Responses to Changes in pH and Temperature

**DOI:** 10.3390/plants5030036

**Published:** 2016-09-07

**Authors:** Suneru P. Perera, Tara C. McIntosh, Janitha P. D. Wanasundara

**Affiliations:** 1 Saskatoon Research and Development Centre, Agriculture and Agri-Food Canada, 107 Science Place, Saskatoon, SK S7N 0X2, Canada; suneru.perera@agr.gc.ca (S.P.P.); tara.mcintosh@agr.gc.ca (T.C.M.); 2 Department of Food and Bioproduct Sciences, University of Saskatchewan, Saskatoon, SK S7N 5A8, Canada

**Keywords:** canola/rapeseed, cruciferin, napin, protein, solubility, secondary structure, tertiary structure, surface hydrophobicity, intrinsic fluorescence, denaturation

## Abstract

The two major storage proteins identified in *Brassica napus* (canola) were isolated and studied for their molecular composition, structural characteristics and the responses of structural features to the changes in pH and temperature. Cruciferin, a complex of six monomers, has a predominantly β-sheet-containing secondary structure. This protein showed low pH unstable tertiary structure, and distinctly different solubility behaviour with pH when intact in the seed cellular matrix. Cruciferin structure unfolds at pH 3 even at ambient temperature. Temperature-induced structure unfolding was observed above the maximum denaturation temperature of cruciferin. Napin was soluble in a wider pH range than cruciferin and has α-helices dominating secondary structure. Structural features of napin showed less sensitivity to the changes in medium pH and temperature. The surface hydrophobicity (S_0_) and intrinsic fluorescence of tryptophan residue appear to be good indicators of cruciferin unfolding, however they were not the best to demonstrate structural changes of napin. These two storage proteins of *B. napus* have distinct molecular characteristics, therefore properties and functionalities they provide are contrasting rather than complementary.

## 1. Introduction

**Figure 1:**
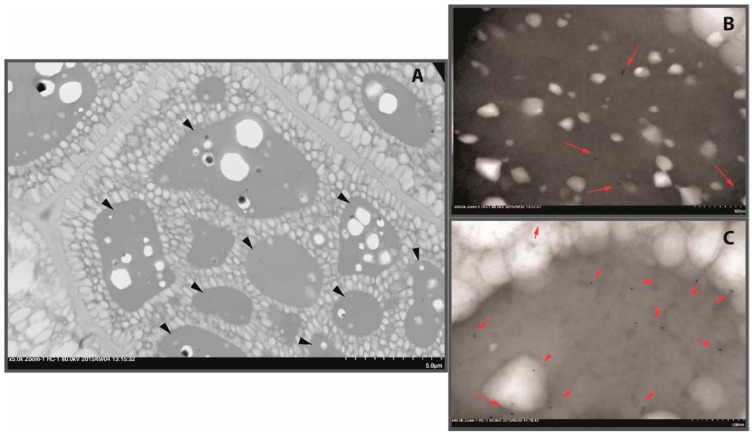
TEM images of *Brassica napus* seed cotyledon cells. (**A**) Oil and protein are stored separately, black arrowheads indicate protein storage vacuoles (PSV) and the areas of PSV visualized with specific antibodies attached with gold particles; (**B**) with anti-cruciferin antibodies; and (**C**) with anti-napin antibodies. Antibodies-attached gold particles are the dark spots pointed out with orange arrows. Both cruciferin and napin are found in the same areas of PSV. (**B**,**C**) are 8× magnified as (**A**). Source: Wanasundara (unpublished).

Similar to many other oil-accumulating eudicot seeds, *Brassica napus* (canola or rapeseed, hereafter referred to as canola) accumulates proteins during the seed-filling stage to restrain N in reduced form for the use of germinating the embryo. Of the total protein accumulated in *B. napus* seed, 60% and 20% of proteins are from cruciferin (11S globulin) and napin (1.7–2S albumin), respectively [1,2]. These proteins are primarily stored in the protein storage vacuoles (PSV) found in cotyledon cells (Figure 1) and cruciferin and napin are the most abundant protein types reported for *B. napus* seed [3,4]. Minor proteins that are of non-storage nature such as oil body proteins (oleosin, caleosin and steroleosin), trypsin inhibitors and lipid transfer protein have also been reported [5,6].

In terms of protein classification on an evolutionary basis, cruciferin and napin belong to two different protein families: cupin superfamily and 2S albumin, respectively [7,8]. Cruciferin (~300 kDa) has a hexameric quaternary structure composed of six subunits or protomers [9,10]. The subunits that form the tertiary structure of cruciferin could be slightly different from each other because of the multiple genes involved in expressing this protein. In *B. napus* cruciferin, the primary structure is composed of 465–509 amino acid residues depending on the expressing gene, therefore five different subunits, namely CRU1, CRU2, CRU3, CRUA and CRU5 have been identified [5,11,12]. Each cruciferin protomer consists of two polypeptides, an *α*-(acidic) and a *β*-(basic) chain.

The structural model of rapeseed procruciferin developed by Tandang–Silvas and group [10] using X-ray diffraction data showed that it possesses 25–27 β-sheets, seven α-helices and three–four 3_10_-helices similar to that of A3B4 protomer in soybean glycinin. Three cruciferin subunits together form each trimer that stack to assemble a hexamer. Recently, Neitz and group [4] analyzed the cruciferin complex in its native state in the PSV of *B. napus* seed and reported that an octameric barrel-like structure of ~420 kDa can be proposed. In crucifers, both 11S and 2S proteins accumulate in the same PSV ([13,14] Figure 1B,C). However, no information is available on how cruciferin and napin associated in the PSV or during formation of molecule complexes.

Similarly, expression of napin is also regulated by multiple genes and 10–16 different napin encoding genes have been identified in *B. napus* [15,16]. Several isoforms, namely Napin-1, Napin-2, Napin-3, Napin-1A, Napin-B and Nap1 with molecular mass ranging from 12.5 to 14.5 kDa have been reported in *B. napus* [17]. Mature napin comprises of a small (short, ~4 kDa) and a large (long, ~9 kDa) polypeptide chain [8] linked together by two inter-chain disulfide bonds, while the large chain possesses two intra-chain disulfide bonds [18].

Proteins are the most useful macromolecule of oil-extracted canola meal in which cruciferin and napin comprise the majority. These proteins are stored in PSV (Figure 1) which are ruptured and possibly mixed and combined with other cellular components during mechanical breakdown of the seed. In order to get full potential of seed protein, its recovery from the cellular matrix of oil-free meal may be essential but poses a technologically challenging task. The inherent differences of constituent proteins and the inter-association with chemical components such as phenolic compounds, simple sugars and phytates found in the cells of cotyledons and the seed coat are identified as the impediments to obtaining canola protein in high purity [5]. It is a known fact that commercially available canola meal is not a suitable substrate for protein product development. Primarily, the changing environment conditions during oil extraction, such as increase in temperature which could be up to 110 °C at the desolventizing step, and also the exposure to non-polar hexane, may cause changes in protein, rendering them less extractable. When the protein recovery processes employed for canola is considered, usually, the aqueous extractions involving pH manipulations that range from basic to acidic have been utilized [5]. Studies by Schwenke and group [9] and Apenten and Folawiyo [19] provide some information on the structural changes of cruciferin and napin due to changes in environmental factors, but not sufficient for benchmarking the structure-related changes that can be followed as markers to understanding the extent of modifications which occur due to extensive processing such as in commercial-scale seed processing. It is expected that such information can be obtained by studying *B. napus* crucifern and napin separately and without any interference from seed matrix components.

The objective of this study was to investigate structural changes of cruciferin and napin of *B. napus* with the changes in temperature and pH. These two structurally different proteins are expected to exhibit distinct properties that are unique for each protein. Obtaining cruciferin and napin of *B. napus* in substantially pure form, characterizing structural features and monitoring these features under different temperatures and pHs were carried out while identifying molecular parameters that can infer structural changes.

## 2. Results

### 2.1. Proteins of B. napus Meal and Their Solubility Behaviour with pH Change

**Figure 2:**
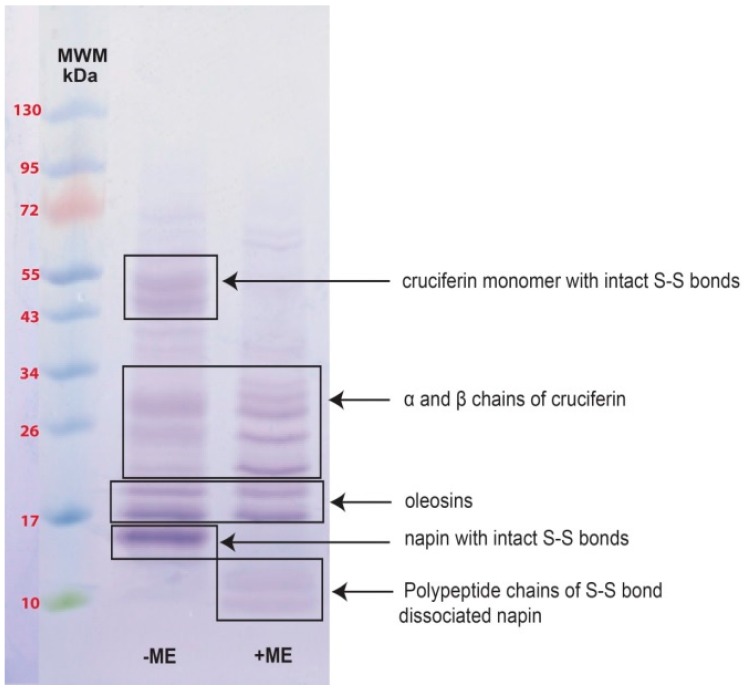
Polypeptide profiles of *B. napus* meal under non-reducing (−ME) and reducing (+ME) conditions in 8%–25% gradient precast gel. MWM (Molecular weight markers). Solid boxes outline polypeptide bands originating from different proteins found in *B. napus* seed and their identification was based on literature [4,5,20].

The de-oiled *B. napus* meal had 42.5% ± 0.6% protein (%N × 6.25) on a dry weight basis. The SDS-PAGE separation of de-oiled meal proteins showed several polypeptides, some with intact disulfide (S–S) bonds that were soluble at pH 8.4 with SDS (Figure 2). The polypeptide bands appeared between 41.0 and 55.0 kDa under non-reducing conditions corresponding well with the monomeric cruciferin subunits that were reported in literature. Several low molecular mass polypeptides were resolved between 14.0 and 32.0 kDa. Under reducing conditions, when S–S bonds were broken, most of the visible peptide bands were in the 9.6–32.0 kDa range. Specifically, the disappearance of 41.0–55.0 kDa and 14.0 kDa polypeptides under reducing conditions indicated involvement of disulfide bonds in stabilizing the association/s of these polypeptides.

**Figure 3:**
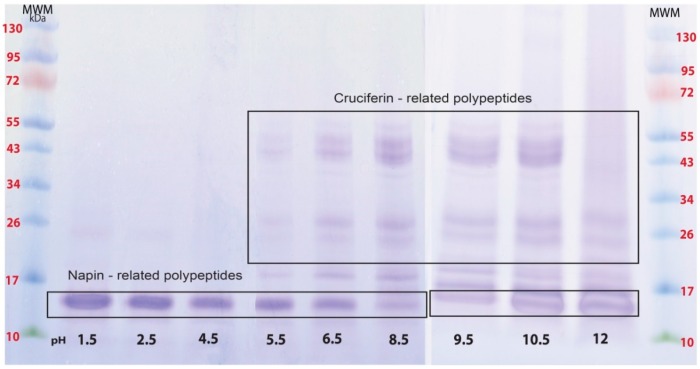
Profile of soluble polypeptides of *B. napus* meal protein with changing medium pH. The pH of extraction medium is indicated under each lane. Each lane contains the same level of protein; SDS-PAGE was under non-reducing conditions, 8%–25% precast gradient gels with molecular weight markers (MWM).

Of the proteins found in *B. napus* meal, differences were seen in the soluble types depending on the medium pH (Figure 3). Proteins of low molecular weight were mainly soluble in pHs between 1.5 and 4.5. Polypeptide patterns of soluble protein between pH 5.5 and 10.5 were similar and large molecular weight polypeptides were found soluble as pH became more basic (Figure 3). Proteins of the meal that became soluble at pH 12 showed disappearance of polypeptide bands in high molecular weight region (Mwt > 34 kDa). However polypeptides between 10 and 34 kDa were identifiable (Figure 3).

### 2.2. Obtaining Cruciferin

**Figure 4:**
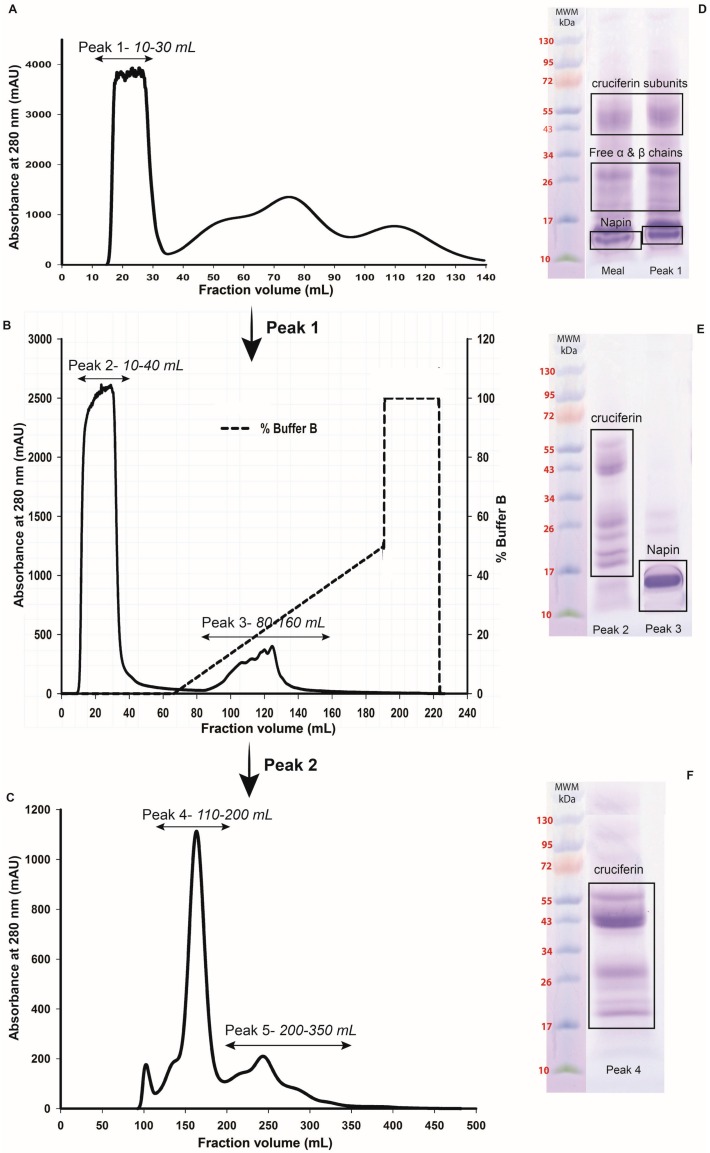
Chromatographic purification steps of *B. napus* cruciferin from pH 8.5, 50 mM Tris-HCl extraction. (**A**) Chromatogram of protein extract separated on Sephadex G-25 Hiprep™ 26/10 desalting column; (**B**) Chromatogram of Peak 1 of desalting column separated on Resource S Cation Exchange Column (CEX); (**C**) Chromatogram of Peak 2 separated on Sephacryl S-300 Hiprep™ 26/10 high-resolution size exclusion column (SEC); (**D**) Polypeptide profiles of *B. napus* meal and Peak 1 obtained from desalting column; (**E**) Polypeptide profiles of Peak 2, Peak 3 and (**F**) Peak 4 obtained from CEC and S-300 SEC, respectively. Polypeptide profiles were obtained under non-reducing conditions using 8%–25% precast gradient gels. Each lane contained approximately the same protein content. MWM: Molecular weight markers.

**Figure 5:**
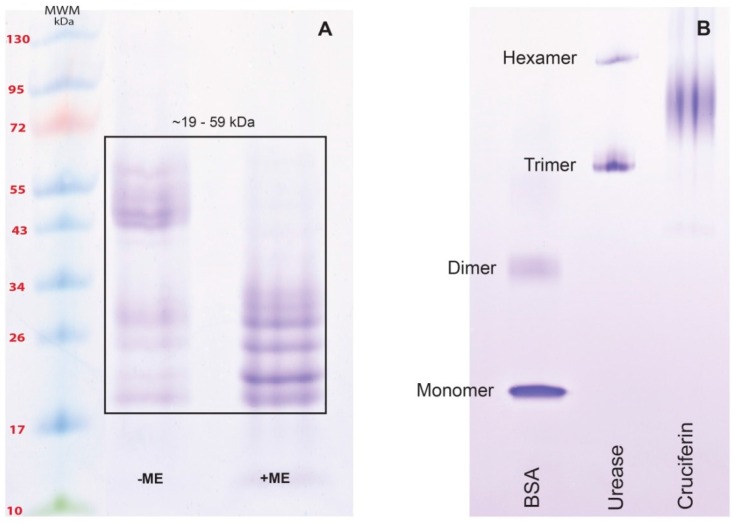
Separation of *B. napus* cruciferin under SDS-PAGE and Native-PAGE. (**A**) Polypeptide profiles of cruciferin under non-reducing (−ME) and reducing (+ME) conditions in 8%–25% gradient precast gel; and (**B**) Native-PAGE separation of purified cruciferin with BSA and Jack bean urease on 8%–25% gradient precast gels with molecular weight markers (MWM).

Canola meal proteins that were soluble in pH 8.5, 50 mM Tris-HCl buffer with NaCl, EDTA and sodium bisulphate as additives were separated as an un-retained peak by size exclusion (desalting) column chromatography (Peak 1 of Figure 4A). The co-extracted pigments and small molecular weight compounds were eluted as non-protein, UV absorbing peak (minor UV absorbance peaks in Figure 4A). The polypeptide profile of Peak 1 was similar to that of the meal polypeptide profile (Figure 2 and Figure 4D) and contained both high and low molecular weight polypeptides. Cation exchange chromatography of Peak 1 proteins resulted in separation of high molecular weight polypeptide-containing proteins in the unbound protein fraction (Peak 2 of Figure 4B,E) and the low molecular weight polypeptide-containing protein in the bound fraction (Peak 3 of Figure 4B,E) at a high salt concentration in the mobile phase. The polypeptide profiles of Peaks 2 and 3 (Figure 4E) indicated that they contained primarily cruciferin and napin, respectively. Since low molecular weight peptides originating from napin were found in Peak 2 (Figure 4E) as a contaminant, the third chromatography step involving a size exclusion chromatography was needed for separating cruciferin. The unbound high molecular weight polypeptide-containing protein (cruciferin) was first eluted (Peak 4 of Figure 4C) and napin eluted slightly late (Peak 5 of Figure 4C), providing cruciferin with much higher purity (Peak 4 of Figure 4F). Reducing and non-reducing SDS-PAGE confirmed characteristic polypeptide bands originating from cruciferin; presence of S–S bonded subunits between 43 and 59 kDa and presence of free polypeptide chains of 19–30 kDa when S–S bonds were dissociated (Figure 5A). Separation of the same protein fraction under native-PAGE indicated that the native conformation of isolated cruciferin was retained (Figure 5B), and oligomeric assembly (can be considered as quaternary structure level) of cruciferin was maintained during this protein purification process. Purified cruciferin did not show any contamination of non-cruciferin protein (Figure 5) and contained >99% protein based on total N analysis (%N × 6.25).

### 2.3. Obtaining Napin

**Figure 6:**
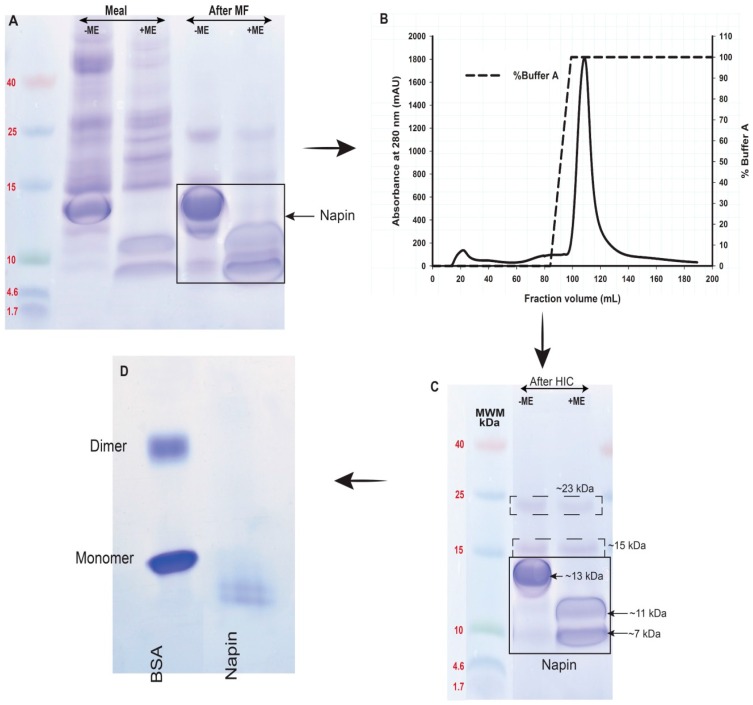
Purification of napin from *B. napus* meal protein extract at pH 3. (**A**) Polypeptide profiles of meal and pH 3 extract after membrane filtration (MF); (**B**) Chromatograms of membrane-separated proteins further cleaned using HiTrap Phenyl Sepharose™ 6 Fast Flow Hydrophobic Interaction column (HIC); (**C**) Polypeptide profiles of napin after HIC; and (**D**) Native-PAGE separation of napin in (**C**). For SDS-PAGE, polypeptide profiles were under non-reducing (−ME) and reducing (+ME) conditions. Precast homogeneous gels of 20% and low range molecular weight markers (MWM) were used.

The three-step chromatographic separation process described here generated both cruciferin and napin (1.6%–2.0% of total meal protein). According to polypeptide profiles of soluble proteins between pH 2.5 and 4.5 (Figure 3), the low molecular weight polypeptides originating from napin dominated; beyond pH 4.5 both napin and cruciferin were soluble. Extraction of *B. napus* meal at pH 3 with NaCl and subsequent membrane separation (2 kDa) eliminated contaminating phenolics and pigments which were of very low molecular weight (Figure 6A,B). These proteins (~13 kDa) consisted of polypeptides of 11 and 7 kDa based upon S–S bond reduction (Figure 6A,C), indicating that the large (11 kDa) and small (7 kDa) polypeptide chains of napin were disulfide linked. Hydrophobic interaction chromatography further polished these proteins (Figure 6B) and resulted in a recovery of 4.9% protein based on the protein level of starting meal. In this purification, two polypeptide bands having 15 kDa and 23 kDa which were not bound by S–S bonds remained along with napin, suggesting possible co-isolation of free acidic chains of cruciferin or some other seed proteins. Native-PAGE profile of purified napin (Figure 6D) confirmed the monomeric nature of napin and the ability to obtain undissociated *B. napus* napin through protein extraction at pH 3 and the subsequent purification process employed. Extraction at pH 3 with 0.15 M NaCl and subsequent purification by membrane separation and HIC provides a less tedious way of obtaining napin protein from *B. napus* meal.

### 2.4. Secondary Structure Features of Cruciferin and Napin and Their Changes Due to Medium pH

**Figure 7:**
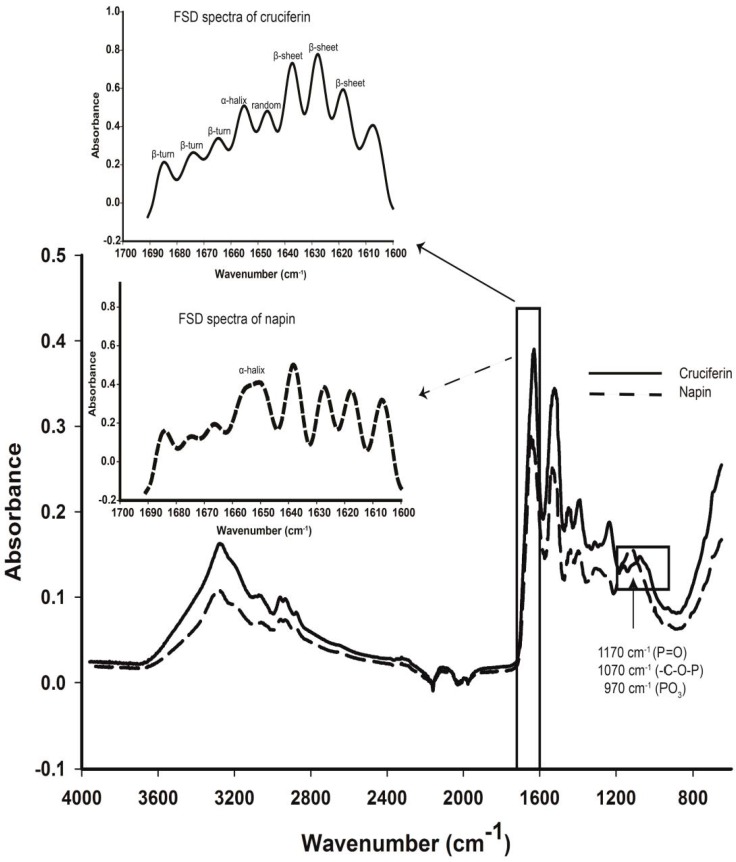
FT-IR spectra of *B. napus* cruciferin and napin. Inset: Secondary structure analysis using amide I region (1600–1690 cm^−1^) Fourier Self Deconvolution (FSD) of amide 1 peak shows secondary structure components and assignment of peaks of the deconvoluted spectra. Deconvolution parameters for cruciferin: Resolution enhancement factor (K) = 2.5, Full width at half height (FWHH) = 14 cm^−1^ and Apodization filter = Bessel. Deconvolution parameters for napin: K = 2.8, FWHH = 18 cm^−1^ and Apodization filter = Bessel. Solid boxes indicate Amide I band and other regions of interest.

Noticeable differences between cruciferin and napin can be observed in the Amide I region and the regions characteristic for PO_3_ (970 cm^−1^), –C-O-P (1070 cm^−1^) and -P=O (1170 cm^−1^) functional groups in the FT-IR spectra of solid proteins (Figure 7). For *B. napus* cruciferin, Fourier Self-Deconvolution (FSD) of Amide I band provided the contents of the secondary structure features, i.e., α-helix, β-sheet, β-turn and random structure. FSD Amide I region revealed predominant β-sheet structure for cruciferin (45.6 ± 0.1) and α-helix, β-turn and random structure contents of 9.4% ± 0.4%, 20.1% ± 0.4% and 3.2% ± 0.5%, respectively. Since napin is known for predominant helical structure [5,8,18], only the α-helix content was evaluated using the FSD process. Napin showed 26.0% ± 0.9% of α-helix content.

When cruciferin in solution was observed for secondary structure components using far-UV-circular dichroism (far-UV-CD), at pH 7, the contents of α-helix, β-turn and random structure were 7.6% ± 0.7%, 20.2% ± 0.9% and 33.1% ± 1.6%, respectively. Assessment of napin gave a typical far-UV-CD spectrum for a predominantly α-helix containing protein, and the estimated α-helix content of 27.5% ± 1.1% and random structure of 26.9% ± 1.4%.

**Figure 8:**
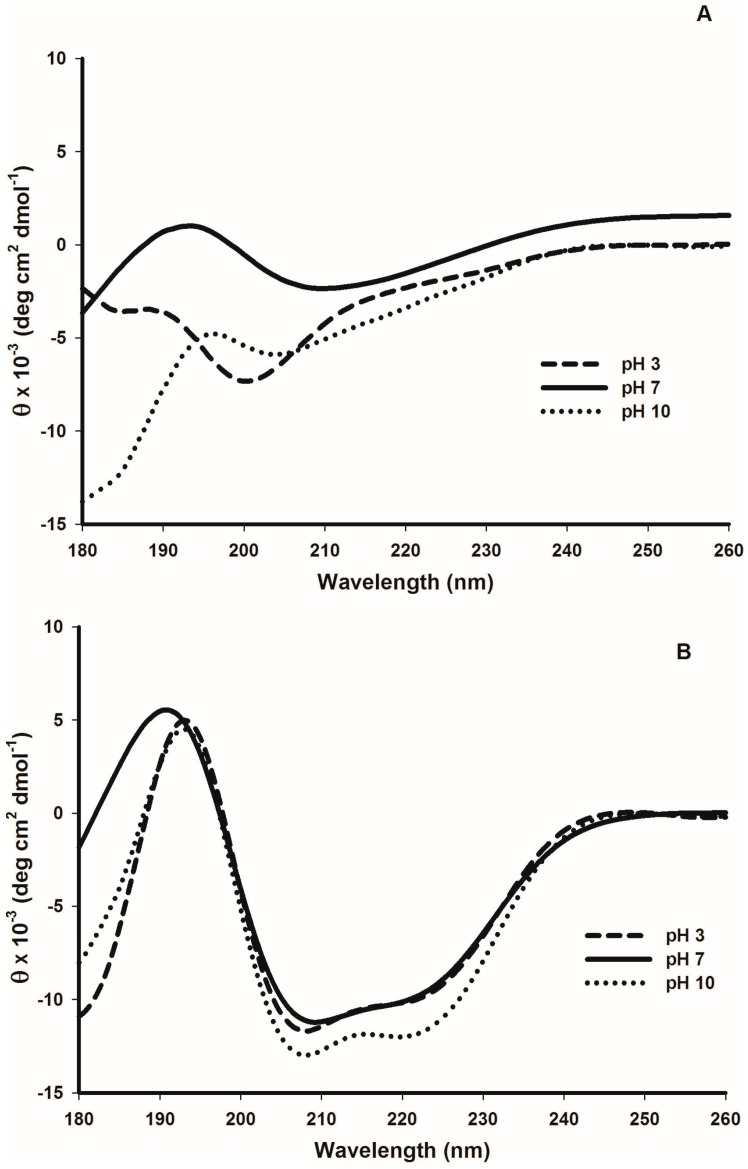
Change in far-UV-CD spectra of purified cruciferin and napin depending on the medium pH. (**A**) Cruciferin; and (**B**) Napin.

The far-UV-CD spectra of cruciferin at different pHs (Figure 8A) showed clear changes in the molecular elipticity (θ), and the contents of α-helix, β-sheet and random structure at pH 3, 7 and 10 indicating possible structural changes occurring with pH change (Table 1). Change in θ as observed at pH 3 indicated the possibility of an unfolded protein structure. The highest α-helix content in cruciferin was obtained at pH 3 (11.6% ± 0.3%) while pH 10 showed the lowest (4.8% ± 0.2%) compared to the values resulting from pH 7 (7.6% ± 0.7%). The highest β-sheet content in cruciferin was observed at pH 7 (39.2% ± 1.9%) and a decreased value was observed as the pH moved to 3 (25.4% ± 3.3%) or 10 (18.4% ± 2.2%). The random structure content in cruciferin increased to 50.5% ± 1.1% at pH 10 compared to that of 33.1% ± 1.6% at pH 7 and 38.0% ± 3.0% at pH 3. Significant changes were observed in the content of β-turns as the medium pH moved from neutral to acidic or basic. However, the values of pH 3 and pH 10 were not that different.

When napin is considered, the spectral features remained more or less similar for the three pHs, showing less sensitivity of secondary structural features of this protein in response to medium pH (Figure 8B). Although the helical content showed a decrease (27.2% vs. 24.1%) at pH 3 compared to that of pH 7 and pH 10 (Table 1), no remarkable difference in random structure content of napin was evident at any pH level tested in this study.

### 2.5. Tertiary Structure Details of Cruciferin and Napin and Their Changes with Medium pH

Evaluation of ANS fluorescence probe binding capacity provides an estimation of surface hydrophobicity (S_0_) of the protein to elaborate on the conformation of protein in a solvent environment [21]. The S_0_ value of cruciferin was much higher (19-fold compared to pH 7) at pH 3 and the lowest was recorded at pH 10 (Table 1). Compared to neutral and basic pH, the S_0_ value of cruciferin at pH 3 was 19-fold and 32-fold higher, respectively, indicating definite and drastic change in the folding state of these proteins.

**Figure 9:**
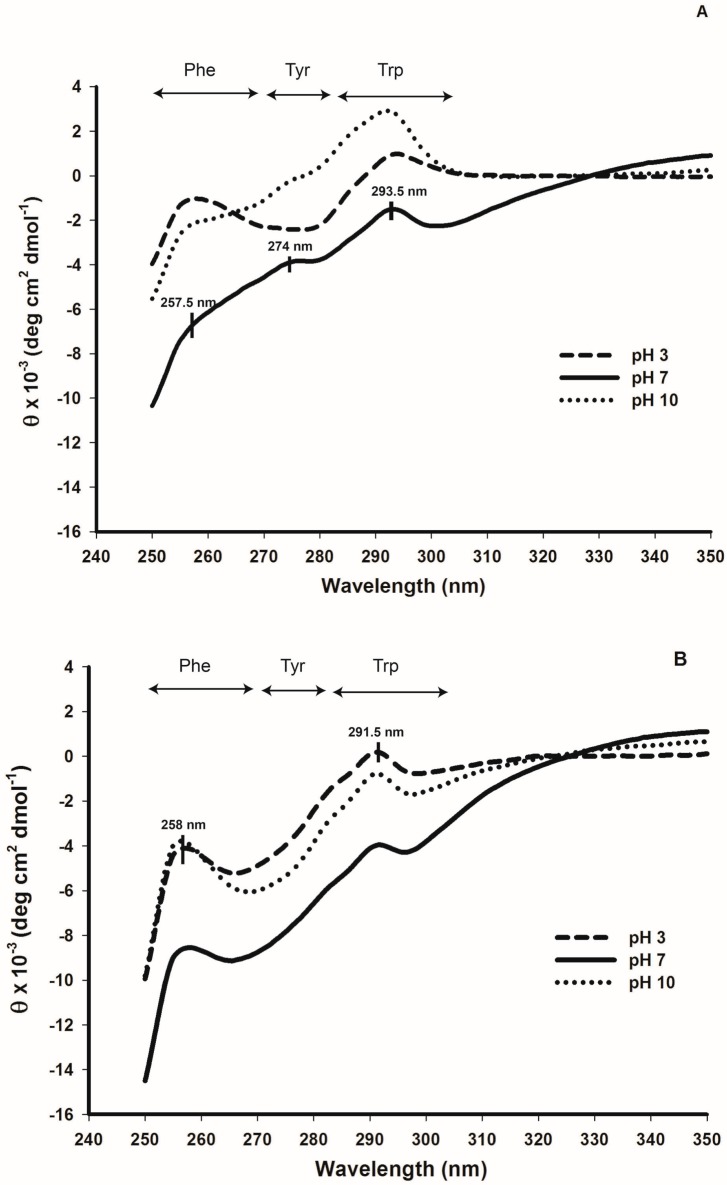
Change in near UV-CD spectra of purified cruciferin and napin depending on the medium pH. (**A**) Cruciferin; and (**B**) Napin. Corresponding regions for hydrophobic amino acid residues are indicated.

The near UV-CD spectra obtained for cruciferin also showed considerable differences with the three pH levels (Figure 9A). At pH 7, for cruciferin, the peaks for Trp (293.5 nm) and Tyr (274 nm) can be easily identified with a weakly appearing peak for Phe (257.5 nm) residues. When the pH of cruciferin moved to 3, distinct peaks corresponding to Phe and Trp residues became visible but not for Tyr. At pH 10, a strong peak corresponding to Trp appeared with fading Phe and Tyr peaks. These changes in the hydrophobic amino acid residue environment indicated pH-dependant changes in the protein tertiary structure. The intensities of Trp suggested that more exposure of these residues at pH 10 compared to that of pH 7 or pH 3.

**Figure 10:**
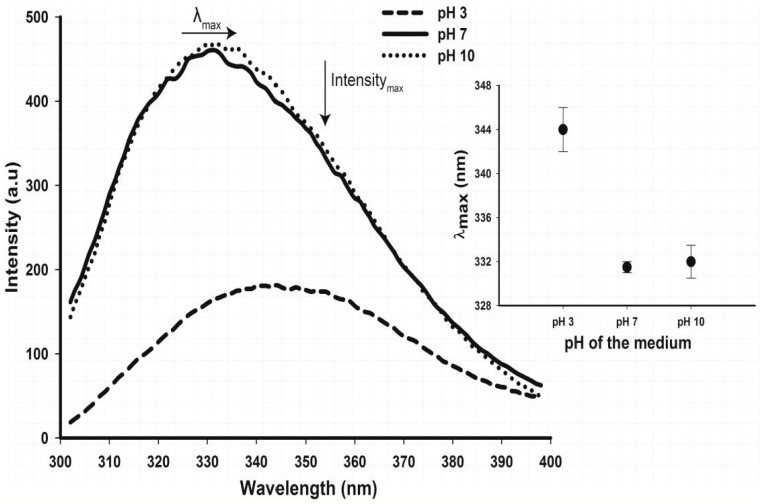
Change of Tryptophan fluorescence of purified *B. napus* cruciferin with pH at ambient temperature (22 °C). All spectra were recorded at an excitation wavelength of 280 nm. Inset: Change of maximum emission wavelength (λ_max_) with medium pH.

**Figure 11:**
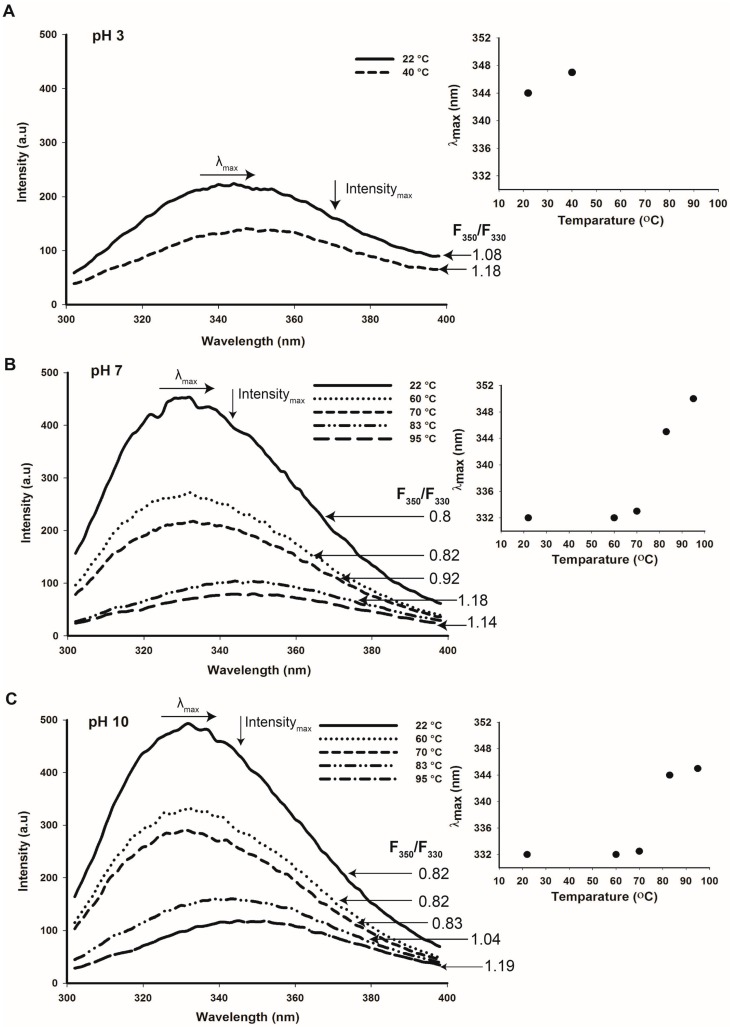
Tryptophan fluorescence of purified cruciferin at pH 3 (**A**), 7 (**B**) and 10 (**C**) with increasing temperatures showing F_350_/F_330_ ratios. Insets: Change of maximum emission wavelength (λ_max_) with temperature at each pH.

The intrinsic fluorescence of Trp residue provided further information on structural changes occurring in cruciferin in these pHs. Although there was no significant change in the maximum fluorescence intensity (F_max_) of cruciferin, the change of pH from neutral to basic and moving to acidic showed a drastic decrease (Figure 10), suggesting possible changes in conformation. This change in the fluorescence spectrum at pH 3 also indicated alteration in the folded state in which the Trp residues are more shielded within the structure when exposed to the bulk solvent. When the fluorescence intensity (F) at 350 nm and 330 nm is expressed as a ratio (F_350_/F_330_), protein folding and unfolding events can be observed. The higher the ratio of F_350_/F_330_, the more unfolded the protein becomes [22]. Among the values calculated for F_350_/F_330_, the maximum of 1.11 ± 0.02 was obtained at pH 3 (Figure 11), indicating cruciferin could be mainly in an unfolded state at pH 3 compared to that of pH 7 and pH 10. Cruciferin exhibited a similar F_350_/F_330_ value of 0.81 ± 0.01 at pH 10 and 7, indicating a change to similar degree of structure unfolding (or folding). This can be further noticed by the red shift in the maximum emission wavelength (λ_max_, Figure 10).

Napin gave a much lower S_0_ value compared to cruciferin at all three pHs (Table 1). Enhanced S_0_ value at pH 3 compared to neutral pH was 12-fold higher. In contrast to cruciferin, a higher S_0_ value for pH 10 compared to neutral pH was observed but it was at a lower magnitude than pH 3 (Table 1). The near-UV-CD spectra of napin showed prominent Trp and Phe peaks at all three pHs (Figure 9B), with a high peak intensity at pH 3 compared to the other two pHs. Napin did not provide consistent results for intrinsic fluorescence (data not shown), confirming very low abundance of Trp and Tyr residues in the structure. Therefore, this technique may be unsuitable for studying conformational changes in napin.

### 2.6. Thermal Stability of Cruciferin and Napin Structure at Different pHs

Distinct thermal transition peaks were observed for cruciferin at both pH 7 and 10 but not at pH 3 (Table 2). At pH 7 and 10, similar values were obtained for cruciferin peak denaturation temperature (T_m_) and enthalpy of denaturation. Onset of protein denaturation occurring at 65–70 °C and ending at 95–100 °C was similar at both these pHs. In contrast, at pH 3, no thermal transition peak was observed for cruciferin.

There were no distinct thermal transition peaks observed for napin at any of these pHs between 30 to 100 °C. When the hermetically sealed DSC pans containing napin were brought beyond 100 °C during DSC runs, reliable thermal transition data for the protein could not be obtained. Dry napin powder also did not give consistent results, but there was a small peak around 56.8 °C and a relatively large peak in the 106–120 °C range appeared for most of the samples. However, the DSC pans containing cruciferin remained stable over the entire temperature ramp from 30 to 130 °C.

Change of intrinsic fluorescence was measured for cruciferin at selected temperatures (T), i.e., ambient T (22 °C), T of onset of denaturation (60–70 °C), peak denaturation T or T_m_ (83 °C) and end T of denaturation (95 °C) at pH 3, 7 and 10 (Figure 11A–C). At both pHs 7 and 10, when the temperature was at T_m_, the F_350_/F_330_ values of cruciferin were higher than 1, indicating a high degree of unfolding with a possibility of further unfolding upon reaching the end of denaturation temperature (Figure 11B,C). As the temperature increased, the maximum emission intensity for cruciferin decreased and the maximum emission wavelength (λ_max_) increased; both these observations provide further evidence on temperature-induced structure unfolding. On the other hand, cruciferin unfolding was minimum at ambient temperature at both pH 7 and pH 10 (Figure 11B,C) and less unfolded (F_350_/F_330_ < 1) at the onset of denaturation. Calculated F_350_/F_330_ value for cruciferin at pH 3 (ambient temperature) was >1 and increased even further at 40 °C (Figure 11A). Similarly, for temperatures above T_m_ gave higher values than 1 for F_350_/F_330_, indicating a high degree of structure unfolding. These results suggest that the structural changes taking place at pH 3 may be somewhat similar to the changes occurring at T_m_ and above.

## 3. Discussion

### 3.1. Obtaining Cruciferin and Napin

Investigation of the *B. napus* meal polypeptide profile confirmed the presence of high and low molecular weight polypeptides (Figure 2) that originate from abundant cruciferin and napin in the seed. Other than these two major proteins, the presence of non-storage proteins, presumably oleosins, was also evident (Figure 2) [4,20,23]. Characteristic polypeptide bands of cruciferin were found between ~40–55 kDa and minor bands in the ~20–32 kDa range that separated under non-reducing SDS-PAGE. The polypeptide bands between ~40 and 55 kDa disappeared upon S–S bond reduction and enhanced intensity of the 20–32 kDa bands [5,6,24]. Working with cruciferin complex obtained from PSV of *B. napus*, Nietzel and coworkers [4] reported a similar polypeptide band pattern in which the α-and β-chain-specific antibodies confirmed that the S–S bond containing bands represent the monomers of cruciferin and the bands that were visible in between the 20 and 32 kDa range as free α-and β-chains which separate during the electrophoresis separation process [25]. Napin, a protein with a molecular weight lower than cruciferin too showed a characteristic polypeptide band of ~14 kDa under non-reducing and ~9–11 kDa chains under reducing conditions similar to previous studies [5,26,27]. Presence of S–S bonds was confirmed by the polypeptides that appeared under reducing conditions representing long and short chains of napin. Napin contains four S–S bonds, two between the chains and two with the large chain [5,6,24].

Separation of cruciferin and napin from total protein extract of oil-free meal required a combination of SEC and CEC steps: first to remove non-protein components and then to separate these two proteins (Figure 4). Napin proteins exhibit basic properties and it is clear from the amino acid composition due to high level of arginine, lysine and histidine compared to other amino acids and the calculated pI of ~pH 11 [1]. In addition, due to the involvement of multiple genes in expressing napins, several isoforms that differ in AA residues [5] are found, possibly giving rise to isoforms soluble in a wide pH range. It is clear that napin is soluble across acidic, neutral and basic pHs, however, only a few isoforms are soluble at pH 8.5 (Figure 3) and allowing a lesser amount of napin available for further purification. However, the selective solubility of napin at pHs ≤ 4.5 provides a suitable strategy for extracting protein without interference from cruciferin. According to Wanasundara and coworkers [28] and Wanasundara and McIntosh [29], extraction of Brassicaceae *(Brassica juncea*, *Brassica carinata* and *Sinapis alba*) seed meals at pH levels as low as 3.0 can capture most of the napin isoforms. Present investigation supports that higher napin yield can be obtained under low pH extraction conditions than at pH 8.5. Further cleaning of this ultra-filtered napin can be achieved through HIC (Figure 6) resulting in napin with >99% protein (N% × 6.25). An identity confirmation study of these proteins using 2DE followed by LC-MS/MS analysis gave the molecular composition of purified cruciferin and napin. Cruciferin (98.9%) is composed of CRU1 (P33523/gene BnC1), CRU2 (P33524/gene BnC2), CRU3 (P33525/gene CRU1) and CRU4 (P33522/gene CRU4) subunits. The remaining 1.1% is contaminated by other proteins including one isoform of napin, i.e., 2SS4 (P17333/gene NAP1) and late embryogenesis abundant protein (LEA 76/P13934). For napin molecular composition, 95.2% is composed of 2SS2 (P01090), 2SS3 (P80208), 2SSI (P24565), 2SSB (P27740/gene NAPB) and 2SSE (P09893) isoforms. The non-napin fraction (4.8%) mainly consisted of the CRU4 subunit followed by CRU3 and LEA 76. It appears that the contaminating polypeptide bands (Figure 6C; 15 and 23 kDa) are possibly free α- and β-chains of cruciferin subunits and LEA 76 protein.

### 3.2. Effect of pH and Temperature on Cruciferin

The cruciferin hexamer (molecular mass 300–360 kDa) is comprised of monomers containing disulfide linked α- (~30–40 kDa) and β- (~20 kDa) polypeptides [7,30]. In each cruciferin monomer, the β-polypeptide chain is buried within the molecule, in contrast to the α-chain that is exposed to the solvent environment [31]. When monomers assemble as a trimer, the inter-chain disulfide bond-containing face (IE face) becomes distinguishable. The IE faces of the trimers pile up via IE face-to-face in assembling the cruciferin hexamer [32,33]. The bonds associated with assembling two trimers together are predominantly of non-covalent nature, such as hydrophobic, electrostatic, hydrogen, van der Walls and hydrogen bonded salt bridges [33], therefore it can be expected that the cruciferin hexameric assembly is sensitive to the changes in pH, ionic strength and any other structure destabilizing conditions in the environment. Cruciferin obtained in this study when separated according to charge-to-mass ratio in the native-PAGE revealed that the oligomeric assembly is conserved (Figure 5B) and the diffused protein band is an indication of isoforms of the protein [34,35].

The predominant β-barrel structure of cruciferin subunits was confirmed by the 45.6% β-sheet structure resulted in for the deconvoluted amide I band of the FT-IR signal. Cupin superfamily proteins have the conserved β-barrel or “*cupa*”. Withana-Gamage [12] studied Arabidopsis (wild type) cruciferin that was obtained through the same purification process and showed 44.1% β-sheet structure content. Low negative elipticity value in far-UV-CD is typical for proteins with low content of α-helical conformation. Using far-UV-CD data, Schwenke et al. in 1983 [9] showed that rapeseed 12S globulin has a low α-helix content (11%) and relatively high content of (31%) of β-conformation. The *α*-helix content of purified cruciferin (9.4%) was comparable with the values reported for cruciferin of Arabidopsis wild type (9.2% [12]).

The effect of medium pH on cruciferin structure in an aqueous environment is evident from far-UV-CD data; a shift in the weak minima at 210 nm and an increase in elipticity observed when pH changed from 7 to 3 pointed out the changing nature of secondary structure features. An increase in the content of β-sheets is considered as an indication of protein aggregation [36,37]. Although cruciferin showed the least solubility at an acidic pH (Figure 3) indicating the protein is aggregated, the decrease in β-sheet content as the pH moved away from neutral did not corroborate with this theory. When the changes in surface hydrophobicity of cruciferin is considered, the maximum S_0_ value was observed at pH 3 which is due to binding of more ANS molecules with cruciferin and providing high fluorescence yield compared to neutral or basic pH (Table 1). The increase in the number of binding sites or protein-ANS binding affinity or change in the surrounding of the binding site is due to protein unfolding that may favor ANS binding [38,39,40,41] and subsequent aggregation due to hydrophobic interaction. Data on cruciferin intrinsic fluorescence and near-UV-CD also confirmed acid-induced unfolding of the native structure. It is highly likely that hydrophobic residues buried in the core of cruciferin trimer are exposed as the hexameric assembly unfolds at pH 3, increasing protein-ANS binding affinity and the number of binding sites, leading to an increase in fluorescence. The in-silico homology modelled Arabidopsis cruciferin structure showed that the IE face of CRUA and CRUB subunits, which are 85.3% and 75.8% homologous, respectively, to *B. napus* procruciferin, contain more hydrophobic residues compared to that of the IA face [12]. The IE face of the two cruciferin trimers that are occluded in the hexameric assembly may have been exposed due to dissociation and caused the increase in hydrophobicity or ANS binding. This may be the reason that the surface hydrophobicity of *B. napus* procruciferin (trimeric) is found to be higher than that of closely packed mature 11S globulin (hexameric) of *Glycine max* [10]. It has also been reported that soybean glycinin (11S globulin) mainly exists as trimeric complexes (7S) at pH 3.8 [42]. Therefore, at pH 3, as a consequence of cruciferin hexamer unfolding, protein becomes more hydrophobic and low in ionized residues causing aggregation. At pH 10, it can be assumed that the environment of protein-binding sites becomes less hydrophobic [19] or a fewer number of hydrophobic sites get exposed, resulting in the lowest S_0_ values producing non-aggregated cruciferin molecules. At pH 7 and 10, as indicated by 0.81 for F_350_/F_330_, cruciferin may exist mostly in hexameric form. When medium pH was brought to 3, the trimeric assembly may predominate, and dissociated subunits may correspond to the F_350_/F_330_ value > 1. Inability to observe a thermal transition peak at pH 3, compared to pH 7 and 10, also confirms the possible structural change that occurs in cruciferin at low pH. Non-significant differences in the peak denaturation temperatures and enthalpy values at pH 7 and 10 also indicate minimum or no-structural change in cruciferin at neutral and basic pHs (Table 2). Fluorescence of tryptophan residue, especially λ_max_, maximum fluorescence intensity and ratio of F_350_/F_330_ or vice versa provide indispensable information on changes which occur in protein structure during unfolding [43,44]. Increase in λ_max_ or red shift upon protein denaturation corresponds with more hydration or availability of tryptophan residues to hydrophilic environment. A major shift in F_330_/F_350_ ratio at T_m_ has been observed and correlated with DSC data of humanized monoclonal antibody (IgG) [43].

The molten globule is a partially folded conformation of a globular protein with near-native compactness, substantial secondary structure, slight tertiary structure and elevated solvent exposed hydrophobic surface area compared to native conformation [45]. With respect to soybean acid-induced denaturation of 12S glycinin, from DSC studies at pH 3, Sung Kim and coworkers [46] have observed denaturation of the protein and showed conserved secondary structure features with an increase in α-helix content (with CD spectra data) similar to what was observed for cruciferin in the present study. According to Bhatty and group [47] rapeseed globulin dissociates into 2–3S components after dialyzing in 6 M urea, especially in acidic buffers below pH 3.6. Schwenke and Linow in 1982 [48] have demonstrated that cruciferin complex exists as 12S at high ionic strength (≥0.5) and dissociates into 7S components when dialysed against water, freeze dried and reconstituted in weakly alkaline water (pH 8.0). It is assumed that the 7S complex is the trimeric half of the hexamer. According to Apenten and Folawiyo [19], acid-induced structural destabilization of cruciferin is reversible. Therefore association and dissociation of cruciferin can be explained as follows.

(1) Native [12S Hexamer]→2×[7S Trimer]↔6×[unfolded subunits]

In this study, we did not investigate the reversible nature of pH-induced unfolding of cruciferin; however, it is quite possible that cruciferin assumes a molten globule state at pH 3. Only few subtle changes of cruciferin structure occur at pH 10, but molecular properties stay similar to neutral pH.

Cruciferin structural features showed progressive changes as temperature increased. Increase in F_350_/F_330_ ratio > 1 and the red shift of maximum emission wavelength of the protein near and above peak denaturation temperature (both at pH 7 and 10) are clear indicators of protein structure unfolding. Not like acid-induced reversible structure unfolding of 12S proteins, structural changes at denaturation temperature are irreversible [19,42].

### 3.3. Effect of pH and Temperature on Napin

The native-PAGE confirmed that napin is in monomeric form with high purity (>99% protein as %N × 6.25, 95.2% based on composition). When *B. napus* napin protein is considered, the three-dimensional structural organization [5,18] shows a globular 4 helical motif with loop regions exhibiting a simple “up and down” topology. Napin has a highly helical secondary structure [18] similar to that of cytochrome C and myoglobin, which are mainly helical [49] and should be treated separately from the model used for cruciferin in secondary structure data analysis. Therefore, different K factor and FWHH were used (Figure 7) to deconvolute napin amide I band as described by Byler and Susi [49]. The deconvoluted amide I band resulted in for napin showed peaks resembling the β-sheets (1627–1638 cm^−1^) and β-turns (1674–1684 cm^−1^) similar to that found in cruciferin (Figure 7). Similar phenomenon were reported for hemoglobin, myoglobin and cytochrome c, at 1627–1638 and 1671–1675 cm^−1^ of amide I region [49]. It is highly unlikely that these proteins contain β-structures; therefore it is possible that these bands are related to some segments associated with the short, extended chains attached to helical cylinders (e.g., residues 79–84, 98–99 and 150–153 in myoglobin), which indeed have neither β-sheets nor β-turns [49,50,51,52]. Using a mixture of napin isomers in which Napin-3 is predominant, Schmidt and group [3] revealed that the secondary structure of canola napin is composed of more α-helices (~48.6% to 59%) and lesser β-sheets (7% to 15%) over the pH range of 3–12. Similar to FT-IR, the deconvolution algorithm used in CD data analysis software has limitations. Hence, the ability to treat the individual protein based on their specific structural features is limited. Previous studies have reported 40%–45% helices and 16%–20% β-sheets [53], 25% α-helix and 38% β-sheets [54] for napin obtained using CD analysis. The secondary structure modelled using the primary amino acid sequence [55] and the solution structure of 2S albumin (RicC3) *Ricinus communis* resolved using NMR [56] confirmed helical napin structure and did not provide evidence for β-sheets. The α-helix content obtained from FT-IR and CD at pH 7 in this study is comparable to the values reported by Krzyzaniak and coworkers [54].

The increase in the fluorescence emission or surface hydrophobicity values at pH 3 (Table 1) and the near-UV-CD spectra (Figure 9) of napin indicated possibility of acid-induced structural changes, although not to the same extent as cruciferin. It is reported that napin exists as a monomer and sometimes as a dimer (Figure 5B). There is no sufficient evidence to conclude that the improvement of hydrophobicity is related to any structural destabilization or denaturation similar to what was observed in cruciferin. Napin did not provide any evidence of thermal transition below 100 °C at any of the pH levels tested in this study. It appears that napin structure is thermally stable at all these pH levels. Krzyzaniak and group [54] have reported a thermal denaturation peak for napin (*B. napus*) at 100.3 °C and 80 °C at pH 6 and 3, respectively. In literature, contradictory values are reported for the heat denaturation of napin. Irreversible unfolding of *B. napus* napin at pH 7 was also reported at 62–63 °C [57]. Jyothi and coworkers [26] observed a broad thermal transition peak (32–70 °C) for napin from *Brassica juncea* at pH 7. Deconvolution of this peak revealed two-stage thermal unfolding of napin with a peak transition at 50.35 °C followed by 62.65 °C, which was reported as reversible. A thermal transition peak at 56.8 °C was observed for solid (~4% moisture) *B. napus* napin in the present study; however, the additional peak observed >100 °C requires further investigation to confirm it is an actual thermal denaturation of napin. This work suggests that napin has a thermos-stable structure that is less affected by the medium pH compared to cruciferin.

### 3.4. Relevance to Seed Protein Utilization

Understanding on the behaviour and property changes of major storage proteins of *B. napus* provides insight into the changes that occur in the protein complement above ambient temperatures and different pH environments. During oil extraction at a commercial scale, canola seed goes through processing steps that involve elevated temperatures of: cooking (~100 °C), pressing (50–80 °C), solvent extraction 60–70 °C) and then desolventization and toasting (up to 110 °C) to recover maximum amount of oil and solvent. Cruciferin and napin, which are located in the PSV, are at low moisture environments with pH levels close to neutral. Cruciferin may be the most sensitive protein for temperature-induced structure changes as it showed thermal denaturation temperatures of 83 °C, which is within the range that canola seed is subjected to during the oil extraction process. Our study shows that at this temperature cruciferin is at unfolded state. However, impact of both these conditions on napin structure is not as drastic as cruciferin. This study also provides insight into changes that can occur in canola SSP during aqueous protein extraction. When neutral or basic pH conditions are used for protein extraction, minimum structure alterations may occur. Usually, protein extracted under these conditions is recovered by low-pH precipitation as a form of concentration step. This drastic pH change may dissociate cruciferin tertiary structure, forming mostly trimers and perhaps monomers to some extent, as indicated by the large change in hydrophobicity values. Subsequent pH adjustment of the low-pH precipitated protein may reverse the trimeric assembly state of cruciferin. Therefore, the abundant protein molecules in canola protein products so obtained are partly acid-denatured, which could be reversed.

Considering the information obtained on cruciferin and napin, *B. napus* seed storage protein complement is composed of two proteins that have quite contrasting structure and structure-related properties requiring special consideration when designing further uses. When they are together, the protein in abundance, obviously cruciferin, may mask the properties of the lesser-abundant protein, napin. However, the opposing nature of structural and structure-related properties may not be advantageous when protein products that are mixtures of cruciferin and napin and their functionalities are considered. These inherent differences of the consisting protein types may explain the differences in functionalities observed between canola protein isolates and legume proteins such as soy [58], in which mostly glycinin (12S legumin) and β-conglycinin (7S vicilin) predominate. Also, it should be noted that *B. napus* contains napin protein which is different than cruciferin in many ways and having unique properties that deserve separate attention for recovery and subsequent uses. The process used in this study and also described by Wanasundara and McIntosh [29] provides a plausible means of recovering napin separately from cruciferin of *B. napus* seed.

## 4. Materials and Methods

### 4.1. Seeds

Greenhouse grown *Brassica napus* (double haploid line-DH12075) was obtained from the protein research lab in Agriculture and Agri-Food Canada, Saskatoon Research and Development Centre, Saskatchewan, Canada. Seeds were stored in a coldroom at 4 °C during the period of analysis.

### 4.2. Meal and Meal Protein

#### 4.2.1. Preparation of Meal

The de-oiled meal was prepared using Swedish tubes with steel balls similar to oil content determination as described in modified Swedish tube method of the AOCS: AM 2-93 [59]. The meal was air dried overnight to remove residual hexane. Total nitrogen content and the polypeptide profile of meal were evaluated according to the method described in Section 4.5 and Section 4.6, respectively.

#### 4.2.2. Types of Protein Soluble at Different pHs

*B. napus* meal and Milli Q water were mixed at a 1:20 (*w*/*v*) ratio and the extraction was performed for 30 min at predetermined pH using a Metrohm 906 Tirando Titrator to maintain constant medium pH (1.5 to 12.0, maximum allowable deviation ±0.05 units) during the extraction. The slurry was then centrifuged at 3500 × *g* for 15 min and the liquid portion was filtered under vacuum through two #4 Whatman filter papers. The polypeptide profile (SDS-PAGE) of resulting extract was analyzed to confirm the types of proteins extracted at each pH level.

### 4.3. Preparation of Purified Cruciferin

Separation and purification of cruciferin from *B. napus* seed meal was performed according to the chromatographic separation procedures explained by Bérot and coworkers [6] with the modification adapted by Wanasundara and group [28].

#### 4.3.1. Extracting Meal Protein

Meal was extracted with 50 mM Tris-HCl buffer (containing 750 mM NaCl, 5 mM EDTA and 28 mM sodium bisulphate at pH 8.5) at ambient temperature with a meal-to-solvent ratio of 1:10 *w*/*v* for 1 h followed by centrifugation at 15,000 × *g* for 10 min. The resulting supernatant was recovered and remaining pellet was re-extracted under same conditions. The supernatants were combined and filtered through Whatman #1 filter paper to remove any floating particles. The extracts were used as fresh as possible and were stored at −20 °C as needed.

#### 4.3.2. Separation and Purification of Cruciferin

As the first step, the extracts were passed through a Sephadex G-25 Hiprep™ 26/10 desalting column (mobile phase: 50 mM Tris-HCl pH 8.5, 1 M NaCl). The resulting protein containing fraction (identified according to absorbance at 280 nm and SPS-PAGE separation) was then dialyzed using 2 kDa molecular weight cut off membrane against deionized water for 48 h at 4 °C and lyophilized. Separation of cruciferin and napin of this protein fraction was performed using a cation exchange column (CEC; Resource S, mobile phase A: 50 mM Tris-HCl, 5 mM EDTA, 0.3% *w*/*v* NaHSO_3_ at pH 8.5, mobile phase B: 50 mM Tris-HCl 5 mM EDTA, 0.3% NaHSO_3_, pH 8.5 containing 1 M NaCl). The unbound protein fraction (cruciferin) which eluted first from the CEC was separated by a size exclusion chromatography (SEC) column (Sephacryl S-300 Hiprep™ 26/10 high-resolution column, mobile phase 50 mM Tris-HCl at pH 8.5 containing 1 M NaCl) for further purification. The bound protein fraction (napin) of CEC was then eluted with a NaCl gradient (5% to 35%). The resulting cruciferin was dialyzed separately similar to the steps described above, then freeze dried and stored at −20 °C until further use. All the chromatographic separation steps described here were carried out using ÄKTA Explorer system (Amersham Pharmacia, Uppsala, Sweden) and the elution of protein was monitored as UV absorbance at 280 nm wavelength. The proteins in each UV absorbance peak were assessed using SDS-PAGE to confirm the identity and the purity. Total nitrogen content of isolated protein was determined as described below.

### 4.4. Isolation and Purification of Napin at Low pH

The procedure described by Wanasundara and McIntosh [29] combined with hydrophobic interaction chromatography was employed to obtain napin. Briefly, napin extraction at pH 3 was performed using *B. napus* meal and Milli Q water at 1:13.5 (*w*/*v*) ratio for 50 min at 30 °C while maintaining pH constant. Protein extract was recovered by centrifuging at 4000 × *g* for 10 min and the supernatant was vacuum filtered with two Whatman #4 filter papers. The meal was re-extracted at pH 3 at 1:7 (*w*/*v*) meal-to-water ratio with 0.15 M NaCl in the medium and protein extract was recovered as before and combined. The protein extract was then separated using a 5 kDa molecular weight cut off and diafiltered to remove salts until chloride ion concentration of the filtrate was <100 µs/cm. The retentate of the membrane filtration was collected and then freeze dried. The separated napin fraction was then further purified using hydrophobic interaction chromatography (HIC) with a HiTrap Phenyl Sepharose™ (GE Healthcare Life Science, Mississauga, ON, Canada) 6 Fast Flow column (mobile phase A: 50 mM Tris-HCl 5 mM EDTA, 0.3% NaHSO_3_ pH 8.5, mobile phase B: 50 mM Tris-HCl 5 mM EDTA, 0.3% NaHSO_3_ at pH 8.5 containing 0.85 M Na_2_SO_4_) using ÄKTA Explorer system. The high salt level of the napin fraction so obtained was removed by passing through a Sephadex G-25 Hiprep™ 26/10 desalting column (mobile phase: Milli Q water). After these purification steps, the resulting napin fractions were dialyzed separately similar to the steps described above, freeze dried and stored at −20 °C until further use. The purity of the napin at each step was assessed using SDS-PAGE and the total nitrogen content was determined as described below. 

### 4.5. N-Based Protein Content

The total nitrogen content (combustion method) of meal and purified protein fractions were determined using AOAC Method 990.03 [60]. To calculate the protein content, nitrogen-to-protein conversion factor of 6.25 was used.

### 4.6. Sodium Dodecyl Sulfite Polyacrylamide Gel Electrophoresis (SDS-PAGE)

The polypeptide profile of meal and purified protein was evaluated by SDS-PAGE under non-reducing (SDS extraction buffer without β-mercaptoethanol: β-ME) and reducing (with β-ME) conditions [61] using precast 8%–25% T gradient gels (except for napin, 20T homogeneous gels were used) adapting the protocol by Wanasundara and coworkers [28]. The samples were prepared in 1.5 mL microcentrifuge tubes using required amount of SDS extraction buffer (5% *w*/*v* SDS in 0.05 M Tri-HCl buffer at pH 8). The final concentration of protein in the SDS extract was 1–2 mg/mL. For reducing conditions, appropriate amount of β-ME was added to make a 5% (*v*/*v*) concentration level. The samples were vigorously mixed in an Eppendorf Thermomixer at 99 °C, 1300 rpm for 10 min. Then the samples were brought to ambient temperature and centrifuged for 10 min at 14,000 *× g*. The protein extracts were loaded on to precast gels with molecular weight standards (4.6 kDa–170 kDa, (Pageruler™ prestained protein ladder, Thermo Fisher Scientific Inc., Burlington, ON, Canada) and processed according to Phastsystem Electrophoresis System-Operating procedure (Pharmacia Phastsystem Electrophoresis System, GE Healthcare Life Science, Mississauga, ON, Canada). Finally, the gel images were processed to obtain the molecular mass estimation of the polypeptide bands using the ImageQuant TL software (GE Healthcare Life Sciences, Mississauga, ON, Canada).

### 4.7. Native Polyacrylamide Gel Electrophoresis (Native-PAGE)

Native-PAGE was performed for purified cruciferin and napin. Proteins were dissolved in 0.1 M phosphate buffer (pH 8.0) containing 0.1 M NaCl to provide 1 mg/mL and 4 mg/mL concentration of cruciferin and napin, respectively. Then the samples were centrifuged at 14,000 rpm for 10 min and the clear supernatant was loaded onto 8%–25% (%T) gradient gel. The electrophoresis was performed according to Phastsystem Electrophoresis System-Operating procedure. Native-PAGE buffer strips (free from SDS) were used to provide non-denaturing conditions. Non-denaturing protein standards (Sigma Aldrich Canada Ltd., ON, Canada, i.e., bovine serum albumin (BSA, 66 kDa monomer and 132 kDa dimer) and urease from jack bean (272 kDa trimer and 545 kDa hexamer) were used as reference molecules.

### 4.8. FT-IR Spectroscopy

FT-IR spectroscopy was used to evaluate the secondary structural details (α-helix, β-sheet, β-turns and random structure) of cruciferin and napin. Briefly, purified protein powder (in dry form) was placed on the ATR diamond surface (Agilent Cary 630 ATR-FTIR analyzer, AgilentTechnologies Canada Inc., ON, Canada) and the sample was pressed against the diamond crystal using the attached pressure clamp. The FTIR spectra were recorded with 4 cm^−1^ resolution and ~30 s measurement time. The secondary structure details were analyzed using Agilent Resolution Pro, version 5.2.0 software and Fourier self-deconvolution (FSD) method of the amide I region (1610–1700 nm) was used to quantify percentage α-helix, β-sheet, β-turns and random structure of each protein.

### 4.9. Circular Dichroism (CD) Spectroscopy

The secondary structural details (α-helix, β-sheet, β-turns and random structure) of isolated proteins were also evaluated using far-UV CD spectra according to method described by Withana-Gamage [12]. A protein solution (1 mg·mL^−1^ of protein) was prepared in 10 mM sodium citrate buffer, sodium phosphate buffer and ammonium buffer at pH 3, 7 and 10, respectively. The far-UV spectrum of the protein solution was then obtained at 25 °C using a PiStar-180 spectrometer (Applied Photophysics Ltd., Leatherhead, UK) equipped with mercury xenon lamp and 0.1 mm quartz cell at 180–260 nm wavelengths using 6-nm entrance and exit slits. The instrument was calibrated with 0.89 mg·mL^−1^ d-(+)-10-camphorsulfonic acid (CSA). Four scans per sample were averaged to obtain one spectrum and the baseline corrected by subtracting buffer spectra. The background corrected spectra were analyzed and molar ellipticity was calculated using CDNN 2.1 software package (Applied photophysics Ltd.). The near-UV (260–320 nm) CD spectra were also obtained using the method described for far-UV spectra. In contrast, samples were introduced to the PiStar-180 spectrometer using 1 cm quartz cell.

### 4.10. Fluorescence Spectroscopy

#### 4.10.1. Intrinsic Fluorescence

Intrinsic fluorescence of the proteins based on the emission spectra of tryptophan residues was evaluated at different pH levels and temperatures. Briefly, the fluorescence emission spectra of protein solution (50 μg·mL^−1^ in buffer solution at 20 °C) were recorded with an Agilent eclipse fluorescence spectrophotometer (Model G9800A, Agilent Technologies, Santa Clara, CA, USA). 10 mM sodium phosphate buffer, pH 7.4, 10 mM ammonium buffer, pH 10 and 10 mM citrate buffer, pH 3.2 were used to provide different medium pH. The tryptophan residues of the protein were excited at 280 nm and emissions scanned from 290 to 450 nm (5 nm excitation and emission bandwidth, medium PMT voltage and factor 5 smoothing using Savitzky–Golay algorithm). The emission spectra of each pH level with increasing temperature (22–95 °C) was recorded and analyzed.

#### 4.10.2. Surface Hydrophobicity

Anionic fluorescence probe 1-anilino-8-napthalensulfonate (ANS) was used to evaluate surface hydrophobicity of cruciferin and napin as described by Withana–Gamage [12] with slight modifications. A stock solution of ANS (8 mM) was prepared at pH 3.0, 7.4 and 10.0 using 10 mM citrate buffer, 10 mM phosphate buffer and 10 mM ammonium buffer, respectively. Then 5 μL of the stock was mixed with 1 mL of protein solution (0.05–0.25 mg·mL^−1^) at each pH level. The solution mixture was incubated for 10 min in the dark and hydrophobic regions were monitored using Cary eclipse fluorescence spectrophotometer (Agilent Technologies) at excitation wavelength of 390 nm and fluorescence emission wavelength 470 nm for cruciferin and 500 nm for napin. To obtain the net fluorescence intensity of protein-ANS conjugate, fluorescence intensity of a protein blank (without ANS) at each concentration was monitored and subtracted from those of protein-ANS conjugate. A linear regression model was fitted for protein concentration vs. fluorescence emission and the surface hydrophobicity index (S_0_) was calculated using the initial slope.

### 4.11. Differential Scanning Calorimetry (DSC)

Thermal properties of purified cruciferin and napin (denaturation temperature and enthalpy of denaturation) were evaluated using a TA Q2000 differential scanning calorimeter (DSC) (TA Instruments, New Castle, DE, USA). Approximately 20 mg of 5% (*w*/*v*) protein (cruciferin) slurry and 10 mg of 10% (*w*/*v*) protein (napin) solution were prepared in 10 mM phosphate buffer (pH 7.4), 10 mM ammonium buffer (pH 10) and 10 mM citrate buffer (pH 3.2). For dry protein analysis, freeze dried protein material was directly analysed without any additives. They were placed into aluminum alodined pans, hermetically sealed with Tzero™ press and subjected to a 30–130 °C temperature ramp at a scanning rate of 2 °C·min^−1^ and 5 °C·min^−1^ for cruciferin and napin, respectively under constant nitrogen purging (flow 50 mL·min^−1^). A hermetically sealed empty pan was used as a reference and results were analyzed using TA universal analysis 2000 software.

### 4.12. Experimental Design and Statistical Analysis

Complete Randomized Design (CRD) was used as the experimental design for the analyses. All the analyses were carried out in triplicates. The results obtained were then analyzed using the General Linear Model (GLM) procedure and Tukey’s test was performed as the post hoc test for mean separation as needed using R statistical software, version 3.2.2 (https://www.r-project.org/).

## 5. Conclusions

The purification processes employed to obtain cruciferin and napin were successful and provide >99% purity (based on % N) with minor contamination from other proteins. Cruciferin and napin exhibit distinctly different secondary and tertiary structural features that show distinct responses to the changing medium pH. Cruciferin structure is sensitive to low pH and showed pH-induced structural destabilization and loss of trimeric assembly causing increased hydrophobicity. Thermal stability of cruciferin is high at pH 7 and 10, whereas it is highly unstable at pH 3. Napin sensitivity to medium pH is much less intense compared to cruciferin, especially at low pH. Thermal stability of napin is high across all pH levels. These contrasting differences of structure and related properties of cruciferin and napin enhance the uniqueness of canola seed protein among other commercially grown oilseeds.

## Figures and Tables

**Table 1:** Secondary structure features ^1^ and surface hydrophobicity of purified cruciferin and napin of *B. napus* at different pH levels.

Protein	pH	α-Helix	β-Sheet	β-Turn	Random	Surface Hydrophobicity
		(%)	(%)	(%)	(%)	(S_0_) ^2^
Cruciferin	3	10.7 ±1.0 ^a^	25.4 ± 3.3 ^a^	26.0 ± 0.7 ^a^	38.0 ± 3.0 ^a^	6666.7 ± 47.2 ^a^
	7	7.6 ± 0.7 ^b^	39.2 ± 1.9 ^b^	20.2 ± 0.9 ^b^	33.1 ± 1.6 ^b^	346.7 ± 6.4 ^b^
	10	4.8 ± 0.2 ^c^	18.4 ± 2.2 ^c^	26.3 ± 0.6 ^a^	50.5 ± 1.1 ^c^	208.0 ± 1.3 ^c^
Napin	3	24.1 ± 0.7 ^a^	NA	NA	26.3 ± 1.3 ^a^	1239.3 ± 19.3 ^a^
	7	27.5 ± 1.1 ^b^	NA	NA	26.9 ± 1.4 ^a^	103.6 ± 3.9 ^b^
	10	27.2 ± 0.7 ^a,b^	NA	NA	25.4 ± 0.6 ^a^	150.4 ± 1.6 ^c^

^1^ From far-UV CD data; Means ± Standard error (SE); ^2^ S_0_ from ANS fluorescence probe; Means followed by same superscript within the same column and same protein type are not significant (*p* > 0.05).

**Table 2:** Thermal denaturation information obtained from DSC analysis for purified cruciferin from *B. napus*.

pH	Denaturation Temperature, T_m_ (°C)	Enthalpy (J/g)	Onset of Peak (°C)	End of Peak (°C)
3	No peak was observed from 30 °C to 130 °C for both the species			
7	83.2 ± 0.8 ^a^	1.1 ± 0.3 ^a^	65–70	95–100
10	84.8 ± 0.2 ^a^	0.9 ± 0 ^a^	65–70	95–100

Means ± Standard error (SE); Means followed by same superscript within the same column are not significant (*p* > 0.05).
